# Two-dimensional speckle tracking echocardiography in goats: repeatability, variability, and validation of the technique using an exercise test and an experimentally induced acute ischemic cardiomyopathy

**DOI:** 10.1186/s12917-020-02277-8

**Published:** 2020-02-14

**Authors:** Aurélia A. Leroux, Marie Moonen, Frédéric Farnir, Stefan Deleuze, Charlotte Sandersen, Hélène Amory

**Affiliations:** 1 Equine Clinic, Department of Companion Animals and Equids, Faculty of Veterinary Medicine, University of Liege, Boulevard de Colonster 20, Bât B41, Liege, Sart Tilman Belgium; 2 Present address: Oniris, 102 Route de Gachet, 44307 Nantes, cedex 3 France; 3 Department of Cardiology, University Hospital of Liege, Medicine Faculty, University of Liege, avenue de l’Hôpital 13, Bât B35, Liege, Sart Tilman Belgium; 4 Biostatistics, Bioinformatics and Animal Selection, Department of Animal Production, Faculty of Veterinary Medicine, University of Liege, Boulevard de Colonster 20, Bât B43, Liege, Sart Tilman Belgium

**Keywords:** Caprine, Cardiac, Strain, Treadmill, Myocardial infarction

## Abstract

**Background:**

Two-dimensional speckle tracking (2DST) technique has been validated in numerous animal species, but neither studies of repeatability nor measurements after exercise or in animals with cardiac disease have been reported in goats. Goats are an attractive candidate for animal models in human cardiology because they are easy to handle and have a body and heart size comparable to that of humans. Therefore, the aim of this study was to validate this technique in goats for further clinical and experimental applications in this species.

**Results:**

This study was divided into several steps. First, a standardized echocardiographic protocol was performed and 5 cineloops of a right parasternal short-axis view at papillary muscles level were recorded three times at one-day intervals in ten healthy adult unsedated Saanen goats to test repeatability and variability of 2DST measurements. Then, the same measurements were performed immediately before and after a standardized exercise on treadmill in seven of the goats, and at 24 h after induction of an experimental ischemic cardiomyopathy in five of the goats, to test the reliability of the technique to assess physiological and pathological changes. Average and regional measurements of radial and circumferential strain and strain rate, radial displacement, rotation and rotation rate were obtained. Comparisons were performed using two-way ANOVA (*p* < 0.05). Caprine 2DST average measurements have demonstrated a good repeatability with a low to moderate variability for all measurements except for the diastolic peaks of the circumferential strain rate, radial strain rate and rotation rate. Segmental 2DST measurements were less repeatable than average measurements. Time effect of two-way ANOVA was significant for anteroseptal segment diastolic peaks measurements, rotation and rotation rate measurements. Overall variability of segmental measurements was moderate or high. Segmental and average peak values obtained after exercise and after myocardial ischemia were significantly different than curves obtained at baseline.

**Conclusions:**

The results of this study are consistent with those previously described in other animal species and humans. 2DST echocardiography is a valid technique to evaluate physiological and pathological changes in myocardial function in goats, despite the technical limitations observed in this species.

## Background

Novel echocardiographic techniques have been developed and allow myocardial function assessment in human and domestic animals [1–3]. Two dimensional speckle tracking (2DST) technique has been used to evaluate regional and global myocardial strain and strain rate. Myocardial strain corresponds to the degree of deformation of a myocardial segment and myocardial strain rate is the rate of deformation [1, 2]. In human medicine, 2DST echocardiography is increasingly included in routine clinical echocardiographic examination of patients. In patients with ischemic cardiomyopathy, this technique is very useful to evaluate the left ventricular (LV) dysfunction and dyssynchrony, to quantify the LV twist pattern, and to predict the response to cardiac resynchronisation therapy [2, 4–6]. Moreover, 2DST during stress testing is used to detect ischemia in asymptomatic patients [7].

In most animal species, 2DST echocardiography remains more frequently used in medical research. Various animal experimental models were developed to study the pathophysiology or therapeutic approaches of human cardiac diseases. Models of ischemic cardiomyopathy, which have been developed by open chest or by percutaneous approach in sheep [8, 9], dogs [10], and pigs [11], currently require 2DST technique to evaluate LV dysfunction. In order to develop a new percutaneous experimental model of ischemic cardiomyopathy in goats, validation of various diagnostic imaging modalities is required. Goats were chosen as an animal model because they are easy to handle and have a body and heart size comparable to that of humans. This makes goats an attractive candidate for animal models in human cardiology research, especially for chronic models relying on measurements in unanaesthetised or exercising animals [12, 13].

The use of 2DST has been described in several domestic animals species such as dogs [14], horses [15, 16], pigs [17], sheep [18], calves [19] and goats [20]. This technique has been recommended to assess LV function in small animals with several cardiac diseases, like chronic mitral valve regurgitation in dogs [21]. In horses, this technique was successfully applied to detect myocardial injuries occurring with various myopathies [22, 23]. In goats, the 2DST technique has been demonstrated to be feasible in standing position and under general anaesthesia [20] but, to the best of the authors’ knowledge, neither repeatability studies of the technique, nor 2DST measurements in exercising animals or in animals with cardiac disease have been reported in goats.

The aim of this study was to validate the 2DST technique in unanaesthetised healthy Saanen goats based on a triple approach: first, a study of repeatability and variability of the technique, second, a study of the reliability of the technique to assess physiological changes induced by a standardized treadmill exercise, and finally, a study of the reliability of the technique to assess pathological changes induced by an experimental myocardial infarction (MI). We hypothesized that the 2DST technique in goats would show a good repeatability for average and segmental 2DST measurements at rest and that the technique would be able to demonstrate significant different values after exercise and after myocardial ischemia compared to baseline, as shown in human patients.

## Results

### Repeatability and variability study

**Fig. 1:**
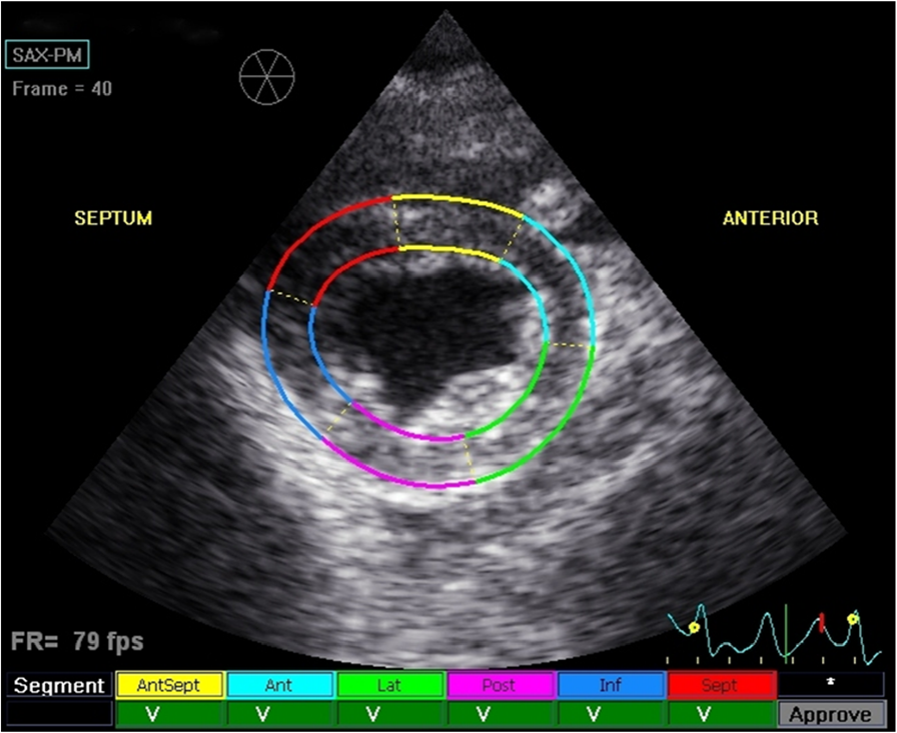
Right parasternal short-axis view of the left ventricle at the papillary muscles level during 2DST analysis. 2DST: two-dimensional speckle tracking; Ant: anterior segment (light blue); Antesept: anteroseptal segment (yellow); Inf: Inferior segment (dark blue); Lat: lateral segment (green); Post: posterior segment (pink); SAX-PM: short-axis view of the left ventricle at the level of the papillary muscles; Sept: septal segment (red)

**Fig. 2:**
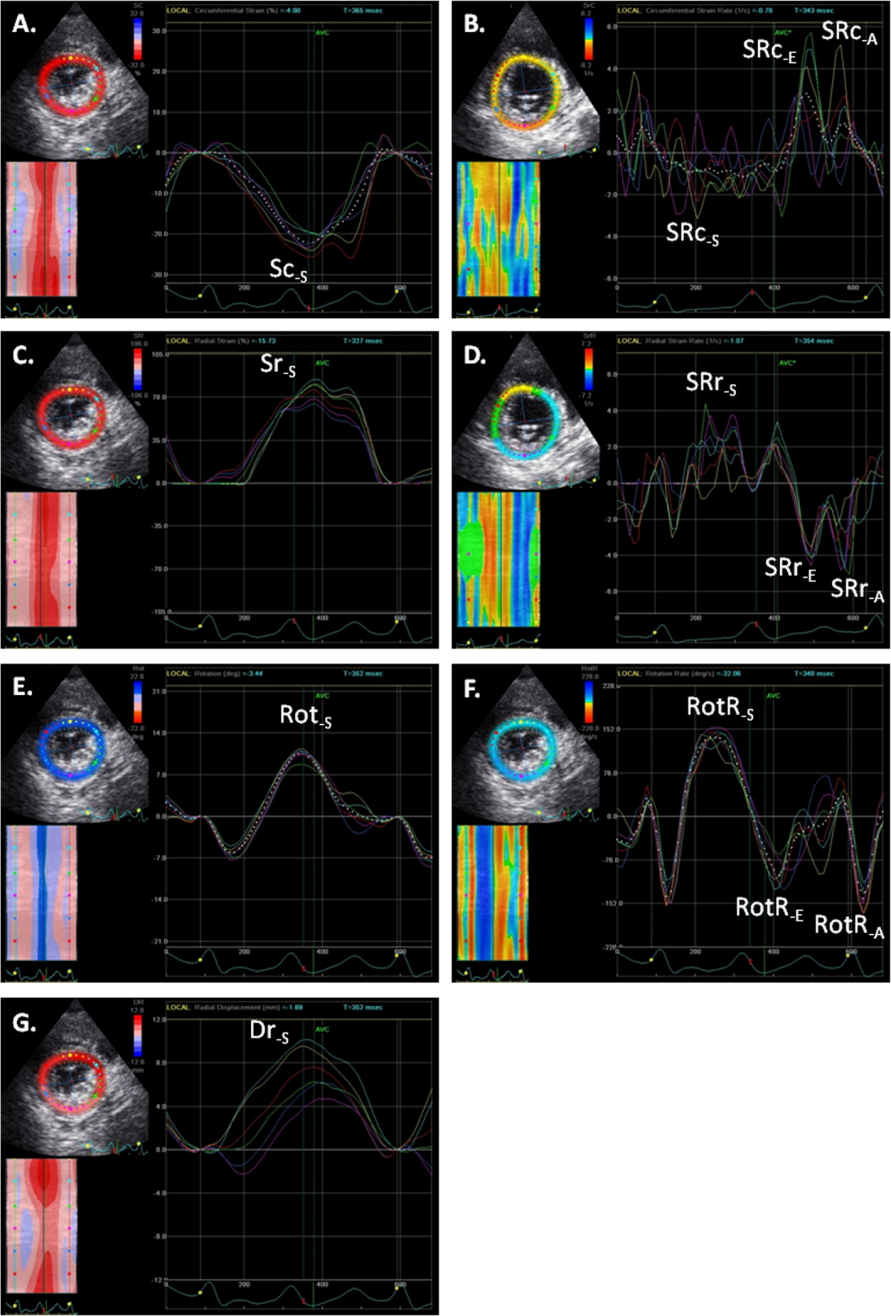
Segmental curves obtained by 2DST analysis for a short-axis view of the left ventricle at the papillary muscles level in an healthy unsedated Saanen goat at rest. **a** Circumferential strain, **b** Circumferential strain rate, **c** Radial strain, **d** Radial strain rate, **e** Rotation, **f** Rotation rate, **g** Radial displacement. -A: measurement during the atrial contraction in diastole; Dr.: radial displacement; −E: measurement during the early ventricular filling in diastole; Rot: rotation; RotR: rotation rate; −S: measurement in systole; Sc: circumferential strain; Sr: radial strain; SRc: circumferential strain rate; SRr: radial strain rate

The echocardiographic protocol repeated three times at one-day intervals on the ten studied goats was easily performed. The mean heart rate during the echocardiographic examination for the repeatability study was 92.7 ± 11.1 beats per minute (bpm). The two-dimensional (2D) image quality was good in all goats and echocardiographic examination was feasible on all 3 days of the protocol. Only four cardiac cycles were analysed in two goats on day 3 because there were not enough recorded non consecutive cycles with an optimal image quality for five analyses. The average frame rate for analysed images was 79.8 ± 10.5 frames per seconds. The software automatically divided the myocardium into six segments according to the human guidelines (Ant: anterior; Antsept: anteroseptal; Inf: inferior; Lat: lateral; Post: posterior; Sept: septal), as shown in Fig. 1. Automated tracking was confirmed as adequate by the software and by visual assessment for 782 of 888 regions of interest (ROI) segments (88.06%). For the remaining segments, a manual correction was necessary because of poor tracking quality at the end of diastole although tracking appeared visually sufficient during the remaining of the cycle. All these segments were manually accepted and included in the analysis. All 2DST variables presented a repeatable curve pattern, as shown in Fig. 2, although RotR curves were more difficult to interpret in three goats. Sc curves were negative for all segments, although curves of the Post segment were more variable in size and morphology than curves of other segments and could present a small positive peak. On the contrary, Sr and SRr of all segments always showed the same tracing and were highly repeatable. Some SRc-S were difficult to distinguish. Dr. and Rot peaks were positive, some being preceded by a small negative peak. RotR-S was highly positive and often preceded by a brief negative peak, whereas the diastolic peaks were all negative.

Repeatability and variability of the segmental and average peak values of each 2DST variable are summarized in Table 1. Concerning segmental measurements, no significant time effect was observed for most of 2DST segmental variables except for the Antsept segment, which was significantly different between day for SRc-E, SRc-A, SRr-E, and Rot-S. For most of the segmental peak, variability was moderate or high although variability of segmental peak values of Sc-S, Sr-S, SRr-S and DR-S between-day and between-cycle CV showed inferior to 25% for at least 2 of the 6 segments (Table 1).

**Table 1:** Repeatability of segmental and average peak values of two-dimensional speckle tracking measurements of left ventricule at the papillary muscles level in ten healthy adult Saanen goats

Variables	Segment	Observed Mean ± SD	95% CI of the mean	Between-cycle CV (%)	Between-day CV (%)
Sc-S (%)	Antsept	−27.26 ± 5.43	− 30.35 – − 23.17	18.38	17.93
	Ant	− 21.85 ± 5.61	−26.08 – − 17.62	24.36	25.31
	Lat	−15.16 ± 5.73	−19.48 – − 10.84	37.79	40.87
	Post	−17.36 ± 6.85 ^a^	− 22.52 – − 12.20	37.99	43.11
	Inf	−23.11 ± 6.21	−27.79 – − 18.43	26.83	26.27
	Sept	−26.84 ± 5.71	− 31.15 – − 22.53	19.83	19.38
	Average	−21.93 ± 3.30	− 24.42 – − 19.44	13.98	14.46
Sr-S (%)	Antsept	72.55 ± 18.01	58.97–86.13	24.14	24.55
	Ant	71.72 ± 21.28	55.67–87.77	28.94	29.62
	Lat	77.25 ± 22.32	60.42–94.08	28.33	29.35
	Post	81.35 ± 22.18	64.63–98.07	26.87	28.33
	Inf	82.37 ± 20.62	66.82–97.92	24.01	25.45
	Sept	80.12 ± 18.25	66.36–93.88	21.51	22.49
	Average	77.56 ± 17.93	64.04–91.08	21.81	22.88
SRc-S (1/s)	Antsept	−2.13 ± 0.47	− 2.48 – −1.78	20.17	21.61
	Ant	−1.93 ± 0.58	−2.37 – − 1.49	27.00	28.45
	Lat	−1.66 ± 0.51	−2.04 – − 1.28	27.78	31.50
	Post	−2.13 ± 0.65	−2.62 – − 1.64	29.35	29.81
	Inf	−2.22 ± 0.61	−2.68 – − 1.76	25.14	26.16
	Sept	−2.29 ± 0.50	−2.67 – −1.91	20.14	22.32
	Average	−2.06 ± 0.29	−2.28 - -1.84	13.22	14.10
SRc-E (1/s)	Antsept	3.58 ± 0.92 ^a^	2.89–4.27	25.21	26.47
	Ant	3.32 ± 1.03 ^a^	2.54–4.10	29.10	33.41
	Lat	2.71 ± 0.88	2.05–3.37	31.54	35.63
	Post	3.25 ± 0.99	2.50–4.00	29.12	31.07
	Inf	2.95 ± 0.87	2.29–3.61	26.97	29.01
	Sept	3.38 ± 0.97	2.65–4.11	26.28	29.36
	Average	3.20 ± 0.56	2.78–3.62	15.85	17.94
SRc-A (1/s)	Antsept	1.75 ± 0.60 ^a^	1.30–2.20	31.03	35.65
	Ant	1.41 ± 0.74	0.85–1.97	46.73	53.29
	Lat	1.05 ± 0.63	0.57–1.53	53.44	57.01
	Post	1.28 ± 0.73	0.73–1.83	55.11	58.34
	Inf	1.51 ± 0.73	0.96–2.06	39.95	47.95
	Sept	1.85 ± 0.74	1.29–2.41	35.00	39.24
	Average	1.48 ± 0.39 ^a^	1.19–1.77	22.25	28.19
SRr-S (1/s)	Antsept	2.88 ± 0.80	2.28–3.48	24.82	24.93
	Ant	3.35 ± 0.97	2.62–4.08	27.10	27.23
	Lat	3.49 ± 0.92	2.80–4.18	24.20	24.65
	Post	3.61 ± 0.91	2.92–4.30	23.26	24.55
	Inf	3.54 ± 0.87	2.88–4.20	22.55	23.00
	Sept	3.11 ± 0.77	2.53–3.69	22.09	23.04
	Average	3.33 ± 0.68	2.82–3.84	18.52	19.07
SRr-E (1/s)	Antsept	−3.23 ± 0.99 ^a^	−3.98 – −2.48	28.26	30.35
	Ant	−3.21 ± 1.19	−4.11 – − 2.31	33.24	37.17
	Lat	−3.30 ± 1.21	−4.21 – −2.39	33.33	38.25
	Post	−3.43 ± 1.16	− 4.30 – −2.56	31.25	35.39
	Inf	−3.43 ± 1.02	−4.20 – −2.66	27.28	31.84
	Sept	−3.29 ± 0.92 ^a^	−3.98 – −2.60	26.81	30.89
	Average	−3.31 ± 0.91	−4.00 – −2.62	26.21	29.35
SRr-A (1/s)	Antsept	−2.28 ± 1.06	−3.08 – −1.48	42.56	46.43
	Ant	−2.45 ± 1.02 ^a^	−3.22 – −1.68	41.41	47.22
	Lat	−2.71 ± 1.02 ^a^	−3.48 – −1.94	34.61	41.29
	Post	−2.84 ± 1.06 ^a^	−3.64 – − 2.04	33.60	42.15
	Inf	−2.88 ± 1.08 ^a^	−3.69 – − 2.07	34.50	41.87
	Sept	−2.60 ± 1.04 ^a^	−3.38 – −1.82	35.41	42.33
	Average	−2.59 ± 0.89 ^a^	−3.26 – −1.92	30.742	37.98
Dr-S (mm)	Antsept	5.90 ± 1.74	4.59–7.21	29.13	30.59
	Ant	7.50 ± 2.28	5.78–9.22	31.36	32.15
	Lat	9.10 ± 1.88	7.68–10.52	20.89	22.19
	Post	8.99 ± 1.91	7.55–10.43	20.22	22.11
	Inf	7.77 ± 2.19	6.12–9.42	26.91	29.07
	Sept	6.46 ± 1.56	5.28–7.64	23.11	25.73
	Average	7.62 ± 0.96	6.90–8.34	11.74	13.26
Rot-S (deg)	Antsept	8.91 ± 2.65 ^a^	6.91–10.91	32.47	34.99
	Ant	10.58 ± 2.43	8.75–12.41	22.55	24.69
	Lat	9.53 ± 2.69	7.50–11.56	28.15	30.17
	Post	7.22 ± 2.95	5.00–9.44	40.29	41.97
	Inf	6.28 ± 3.08	3.96–8.60	45.29	47.76
	Sept	7.01 ± 2.75 ^a^	4.94–9.08	36.70	40.65
	Average	8.23 ± 2.37	6.44–10.02	27.39	29.91
RotR-S (deg /s)	Antsept	97.89 ± 26.94	77.58–118.20	25.47	26.64
	Ant	113.01 ± 22.49	96.05–129.97	18.51	21.60
	Lat	112.02 ± 27.68 ^a^	91.15–132.89	24.48	25.88
	Post	93.11 ± 25.27 ^a^	74.06–112.16	27.93	30.29
	Inf	83.43 ± 25.99	63.83–103.03	30.35	31.17
	Sept	85.30 ± 25.72	65.91–104.69	30.71	31.12
	Average	97.47 ± 20.89	81.72–113.22	20.36	22.17
RotR-E (deg /s)	Antsept	−73.18 ± 31.71	−97.09 – −49.27	42.78	46.40
	Ant	−77.62 ± 30.53	−100.64 – −54.60	36.66	40.06
	Lat	−83.60 ± 34.68 ^a^	− 109.75 – −57.45	41.87	47.22
	Post	−79.21 ± 37.71	− 107.64 – −50.78	46.72	49.43
	Inf	−80.36 ± 36.85	−108.14 – −52.58	46.88	47.99
	Sept	−73.94 ± 33.03	−98.84 – −49.04	47.81	47.37
	Average	−77.98 ± 26.08	−97.64 – −58.32	31.49	35.50
RotR-A (deg /s)	Antsept	−55.94 ± 29.45	−78.15 – −33.73	50.14	51.96
	Ant	−60.62 ± 28.93 ^a^	−82.43 – −38.81	44.53	47.35
	Lat	−67.85 ± 33.02	−92.75 – −42.95	47.21	50.23
	Post	−62.27 ± 33.96	−87.88 – −36.66	55.86	58.53
	Inf	−55.57 ± 31.08	−79.00 – −32.14	54.72	58.43
	Sept	−50.52 ± 29.02	−72.40 – −28.64	58.75	58.81
	Average	−59.22 ± 23.09	−76.63 – −41.81	40.61	42.58

^a^ Day-effect significantly observed with *p* < 0.05 (two-way ANOVA)

*-A* Measurement during the atrial contraction in diastole, *Ant* Anterior segment, *Antsept* Anteroseptal segment, *CI* Confidence intervals, *CV* Coefficient of variation, *SD* Standard deviation, *Dr.* Radial displacement, *−E* Measurement during the early ventricular filling in diastole, *Inf* Inferior segment, *Lat* Lateral segment, *Post* Posterior segment, *Rot* Rotation, *RotR* Rotation rate, *−S* Measurement in systole, *Sc* Circumferential strain, *Sept* Septal segment, *Sr* Radial strain, *SRc* Circumferential strain rate, *SRr* Radial strain rate

Average peak values of the 2DST measurements corresponding to the mean of the 6 ROI peak values showed a good repeatability since no effect of the day was observed on these measurements and variability of most average peak values was low to moderate except for SRc-A, SRr-E, SRr-A, Rot-S, RotR-E and RotR-A (Table 1).

### Stress echocardiography

**Fig. 3:**
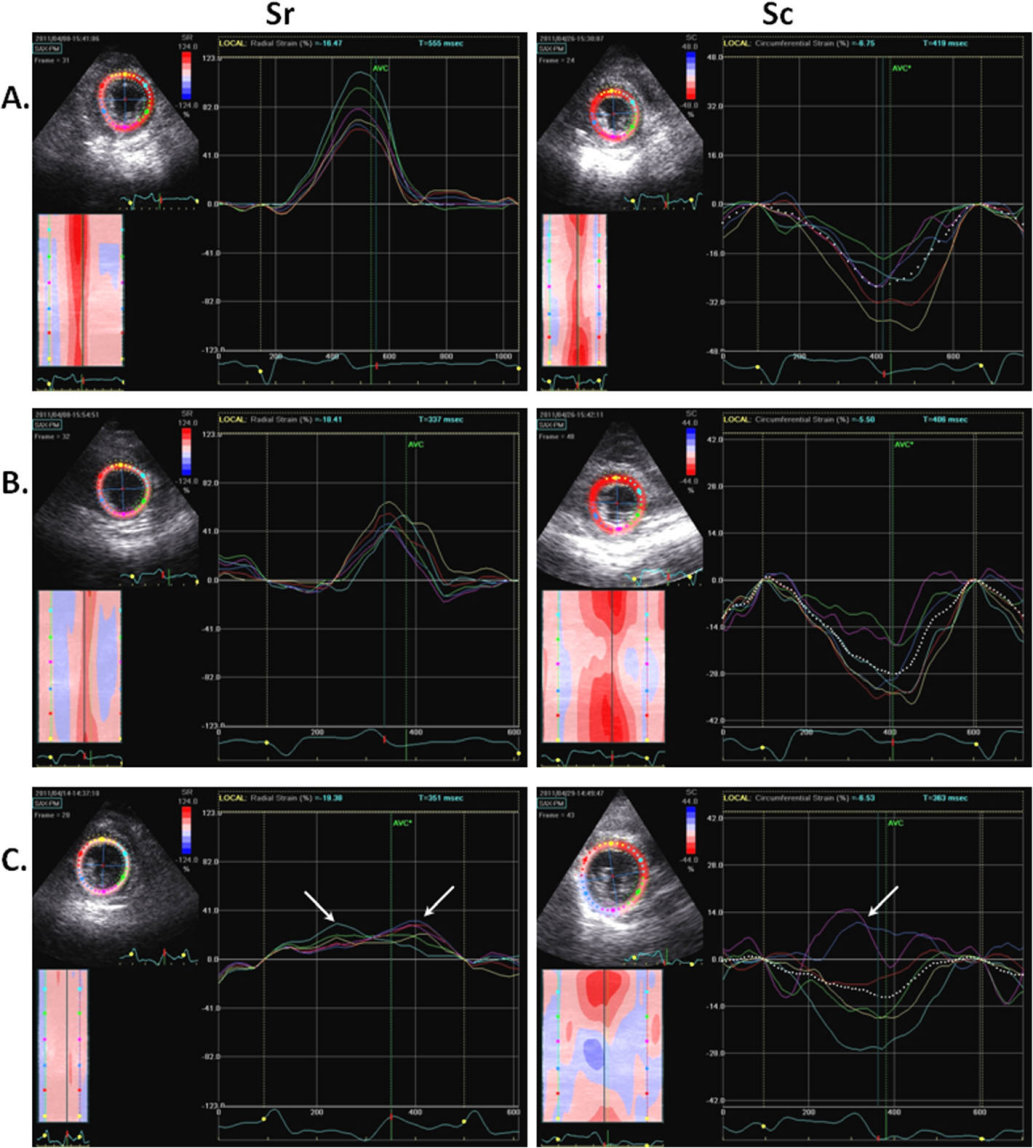
Sr and Sc segmental curves obtained by 2DST analysis in a Saanen goat at rest, just after a standardized exercise test on treadmill, and 24 h after experimental induction of an ischemic cardiomyopathy. **a** Synchronous segmental Sr and Sc curves at rest in a healthy Saanen goat. **b** Synchronous segmental Sr and Sc curves in a healthy Saanen goat just after exercise. **c** Segmental Sr and Sc curves in a Saanen goat at 24 h after occlusion of the terminal branch of the circumflex coronary artery: posterior, inferior, and septal segments showed a severe left ventricular dyskinetic movement (white arrows) characterized by timing differences for Sr systolic peak and a systolic peak inversion for Sc. 2DST: two-dimensional speckle tracking; Sc: circumferential strain; Sr: radial strain

**Fig. 4:**
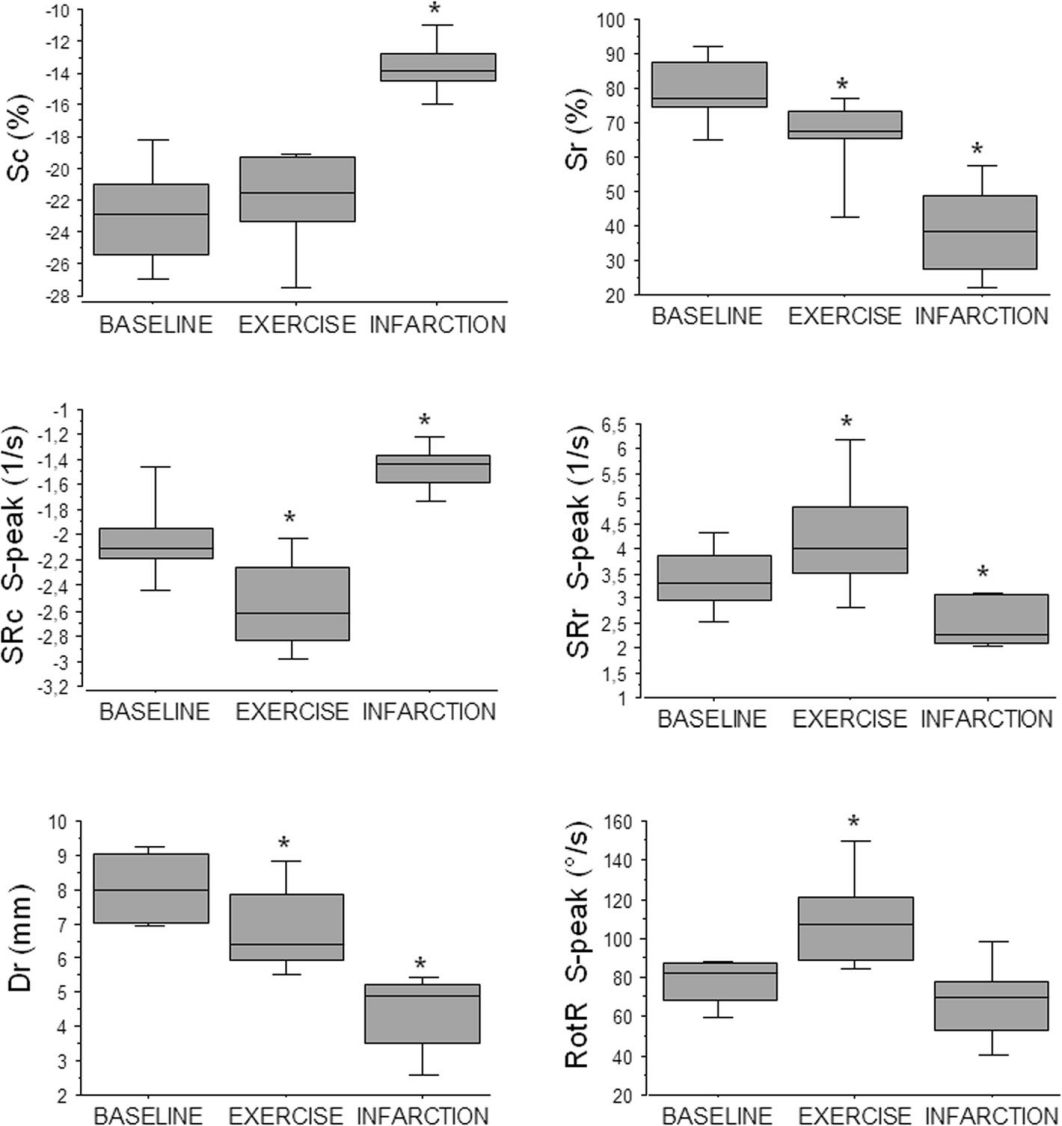
Average systolic peak values of 2DST measurements of left ventricle at the papillary muscles level in goats at baseline (10 goats), immediately after exercise (7 goats) and 24 h after an experimentally-induced myocardial infarction (5 goats). * results significantly different between measurements obtained at rest and immediately after exercise in 7 goats and between measurements obtained before and after myocardial infarction in 5 goats (two-way ANOVA; *p* < 0.05 considered significant). 2DST: two-dimensional speckle tracking; Dr.: radial displacement; RotR: rotation rate; Sc: circumferential strain; Sr: radial strain; SRc: circumferential strain rate; SRr: radial strain rate

Stress echocardiography was easily implemented. Selected goats learnt rapidly how to run on the treadmill and were sufficiently easy to handle to undergo the echocardiography just after the exercise test. Images were of good quality in six of the seven goats. Only 3 to 4 cineloops with the target HR (between 130 and 140 bpm) were kept and analyzed. All the 162 obtained ROI segments were analysed. The mean HR before and after exercise were 90.1 ± 5.1 bpm and 132.1 ± 3.9 bpm, respectively. All segments were judged normokinetic and the obtained curves had similar shapes to the curves obtained at rest, except for post-exercise diastolic peaks of SRr, SRc and RotR, which were fused in 4.73% of the curves (23 of the 486 analysed SRr, SRc and RotR curves). Since fused diastolic peaks did not allow measurements of E and A peaks, these curves were excluded from the analyses. The post-exercise curves for Sc and Sr showed reduced amplitude, but still demonstrated the same curve pattern, as shown in Fig. 3. Segmental peak values obtained after exercise were significantly different than curves obtained at rest, except for Sc, Rot and diastolic peaks SRc-E, SRr-E, SRr-A and RotR-A. The same results were observed for average peak values (Fig. 4).

### Post-ischemic echocardiography

Echocardiography at 24 h after MI creation was easily performed. However, as all goats presented an acute ischemic cardiomyopathy at that time, premature ventricular complexes were observed in all goats but care was taken to exclude these cardiac cycles from analyses. HR during post-ischemic examination was significantly higher (133 ± 2.8 bpm) than before MI (80.8 ± 2.1 bpm). Images were of good quality in four of the five goats. Five cardiac cycles were analysed for these four goats and four cardiac cycles were analysed for the fifth goat. All 144 obtained ROI segments were analysed. Due to high HR, diastolic peaks of SRr, SRc and RotR were fused in 16.43% of the curves (71 of 432 analysed SRr, SRc and RotR curves). Since fused diastolic peaks did not allow measurements of E and A peaks, these curves were excluded from the analyses. Global LV dysfunction was observed: all regional strain and strain rate peak values were significantly reduced as compared to measurements at baseline (before MI creation). Comparison of average peak values at rest, after exercise and after infarction is shown in Fig. 4. The measurements obtained at 24 h after MI showed a qualitatively severe modification of the curve patterns after MI, especially for the Sept, Post and Inf segments. 2DST curves for Sc and Sr, obtained at baseline, after exercise test and at 24 h after MI, are illustrated in Fig. 3. These results were compatible with a severe LV dyskinetic movement characterized by subjective timing differences for Sr systolic peak and a systolic peak inversion for Sc.

## Discussion

The results of this study demonstrate that 2DST echocardiographic technique can be successfully used not only in unsedated healthy Saanen goats, but also after a treadmill exercise and following induction of pathological changes. Obtained curves of Sc, Sr, SRc, SRr and Dr. had characteristics similar to those previously obtained in goats and other animal species [14–16, 19, 20]. Rot and RotR curves were often difficult to interpret but the same curve pattern was observed in all goats, unlike what has been described in horses [16]. Quantitative measurements of 2DST variables were similar to those previously obtained in goats [20] and confirmed the reliability of 2DST measurements in this species. However higher values of Sr-S have been obtained compared to the previous study performed on goats [20]. This difference could be explained by the images acquisition (different views and operators), by the offline 2DST analysis (different positioning of the ROI) or more probably by the studied animals, since seven of the ten goats included in the present study were accustomed to perform daily exercise on treadmill and could have developed a higher systolic LV performance, consistent with higher values of Sr-S.

The caprine 2DST echocardiographic measurements did not show any significant time effect, except for diastolic peaks values of SRc, SRr, and RotR and for the Antsept and Post myocardial segments. Moreover between-cycle and between-day variability of 2DST measurements in goats was low to moderate for average systolic peaks of Sc, Sr, SRc, SRr and DR, which allowed considering these measurements as repeatable. Moderate to high variability was observed for average Rot, RotR, diastolic peaks of SRc and SRr and for all segmental peak values. Similar variability has been previously observed in calves and in horses [16, 19], while low to moderate variability has been reported in dogs and humans [14, 24]. Because of the overall moderate to high variability obtained in this study, 2DST measurements in goats should be interpreted with caution. In the present study, Sc curves had a higher variability than Sr curves, as previously shown in calves [19], in horses [15, 16] and, to a lesser extent, in humans [24]. As Rot and RotR peaks values showed a high variability and a questionable repeatability, their use should preferably be avoided like in horses and calves [16, 19]. Measurements of torsion deformation described in human and dogs [25, 26] might therefore not be feasible in goats. The high variability of 2DST measurements in goats can be explained by several effects: image recording effects (since each measurement was made on a different video loop), software analysis errors (since a delay in tracking myocardial pixels was often present at the end diastole), and measurements errors themselves [27]. Many artefacts were also created by the breathing and were related to heart movement inside the chest from loop to loop. A poor tracking quality at the end of diastole has also been previously observed in calves [19]. This might be due to an image recording effect, software analysis errors or a decrease of image resolution at the end of diastole. However, like in calves, this software analysis deficiency did not seriously alter the tracking quality, which appeared sufficient to allow 2DST analysis [19]. Moreover the overall observation that the between-day CV does not differ much from the between-cycle CV might indicate that most variability observed in this study is caused by between-cycle differences and not by day-to-day differences, since between-day CV and between-cycle CV were calculated from the same measurements of single cycles.

Observation of an exercise effect on 2DST values in goats confirms that this echocardiographic technique is interpretable and sufficiently sensitive to measure physiological cardiovascular changes. Stress echocardiography was easily performed in goats and the exercise induced by the standardized exercise treadmill test enabled the observation of a significant difference between values at rest and just after exercise. These results are in agreement with those obtained in horses [28] and humans [7]. However, in the present study, the requirements for determining the end of the exercise test were difficult to choose. As echocardiographic measurements were shown to vary strongly with HR and with exercise intensity, standardized exercise tests were developed in horses [29, 30] and humans [31]. This is not the case in goats, although different exercise tests have already been performed in this species for other reasons than stress echocardiography [13, 32]. Moreover, unlike in humans, echocardiography could not be achieved during an exercise test in animals, thus stress echocardiography in animals was performed just after the end of exercise [28, 30]. Considering the significant effect of HR on echocardiographic measurements, this variable was used to standardize the 2DST measurements after exercise [28]. The duration of the exercise, as well as the speed and the slope, were chosen in accordance with a previous study [32]. The targeted post-exercise HR was subjectively chosen in order to have a significant difference between baseline and post-exercise HR. Images were recorded only when HR was between 130 and 140 bpm. HR of most goats was within this range immediately after the exercise test, however two goats showed a higher HR (150 to 180 bpm), requiring recuperation time for HR to decrease. Therefore, images matched either with immediate post-exercise peak HR or with recuperation time. To minimize this problem, images were recorded within 2 min post-exercise, as previously recommended [29, 30]. Just after exercise, Sr measurements significantly decreased, SRr and SRc measurements significantly increased whereas Sc measurements at rest and after exercise failed to show a significant decrease induced by exercise. These results are consistent with those previously observed in horses, pigs and humans [5, 28, 33] since decreased Sr may mirror the post-exercise decrease in ejection fraction and stroke volume, and increased SRr and SRc suggest increase in regional and global myocardial contractility. The lack of significant difference in Sc measurements could be due to the higher variability of Sc compared to Sr in the studied goats.

This study also showed that 2DST echocardiography in goats could be applied not only to physiological, but also to pathological conditions. Although 2DST measurement variability was high in this study, results obtained in the case of acute ischemic cardiomyopathy were convincing. Goats with acute ischemic cardiomyopathy presented a severe decrease of average and segmental peak values consistent with depression of the myocardial contractility and the LV function as previously reported in humans [34]. 2DST echocardiography also allowed to suspect infarct location (Post, Inf, and Sept segments) since infarcted segments showed severe myocardial dysfunction and dyssynchrony, similar to that observed in human patients suffering from ischemic cardiomyopathy [4, 6]. Unfortunately, average and regional myocardial synchrony was only assessed qualitatively by observing the curve patterns but was not assessed quantitatively with time measurements as recommended in patients that are candidates for cardiac resynchronization therapy [6]. Based on a qualitative study of the curves, Sc looked less severely dyssynchronic than Sr, but presented a curve inversion for ischemic segments, reflecting a severe myocardial dysfunction at that level [4, 35]. During this protocol, 2DST analyses were limited to the interpretation of LV systolic function, since diastolic E and A peaks of SRc, SRr and RotR were difficult to interpret and were regularly fused, as previously described in goats for mitral and tricuspid flows with pulsed wave Doppler echocardiography [36]. A difference in 2DST sensitivity to ischemia has been described in humans between Sr, Sc and longitudinal strain, the last two presenting alteration earlier than Sr [34], whereas Sr was reported to be more sensitive to predict response to cardiac resynchronization therapy [4]. Unfortunately, this difference of sensitivity to ischemia between Sr and Sc was not examined in the present study but might be interesting to test in further studies to confirm the similarity between natural occurring MI in human and the present experimental model.

This study carried several major limitations. The first one was the missing quantitative time measurements for LV dyssynchrony evaluation, as previously discussed. Another major limitation of the study was the choice of recorded image orientation for 2DST analysis. Analyses were performed only on short axis views of the LV, while short axis and long axis views were used in humans, dogs, horses and in the previous study in goats [3, 20, 23, 28, 35]. In humans, the apical 4-chambers long axis view is often used for colour flow and pulsed wave Doppler echocardiography, for tissue Doppler imaging, and for acquisition of myocardial longitudinal strain [2, 35]. In this study, a short axis view at the papillary muscles level was chosen because this view was easier to obtain and to standardize than a long axis view, and because the image quality of this view was generally higher than that of other views in goats [37]. Moreover, this view allowed obtaining Sr and Sc, which remain good methods to assess LV dyssynchrony in humans [4, 6].

## Conclusions

In conclusion, as previously described [20], 2DST echocardiography was applicable in goats and showed a good repeatability but a moderate to high variability. Physiological and pathological changes of myocardial function in goats can be assessed by 2DST echocardiography. Therefore, 2DST technique could be very useful to evaluate average and regional myocardial function in an experimental goat model of ischemic cardiomyopathy and in naturally occurring myocardial diseases in goats like clinical cases of white-muscle disease or cardiotoxic plants poisoning. Further studies are required to assess quantitatively the LV dysfunction and dyssynchrony and to confirm its use in clinical cases and in research studies.

## Methods

### Animal preparation

This experimental protocol was part of a larger study and followed the guidelines of the ethical use of animals of the University of Liege (approval number 655), in accordance with the European directive 2010/63/EU. Ten adult nulliparous female Saanen goats were included in this study. They were aged 22 to 28 months (mean age: 24.7 ± 2.1 months), they weighed 51 to 80 kg (mean body weight: 65.1 ± 8.3 kg) and they were accustomed to handling. Included animals were bought from a private breeding farm producing goat milk located less than 20 km from the University. All animals were considered healthy based on medical history and the absence of abnormalities on physical examination, cardiac auscultation, electrocardiography, haematology and standard biochemistry panel. A complete standard 2D, time-motion mode and Doppler echocardiography (from right and left side) was performed on each goat before starting the protocol to ensure that the studied goats were free of any cardiac disease. Studied goats were housed in groups of three or four in an enclosed barn with natural ventilation, in accordance with appendix A of the European convention for the protection of vertebrate animals used for experimental and other scientific purposes (ETS NO. 123). All goats were fed with hay ad libitum. Seven of the ten studied goats were randomly chosen to be accustomed to perform a standardized exercise on treadmill, which consisted of walking on a treadmill (Domyos TC290, Decathlon, Villeneuve d’Ascq, France) for 6 min at 4 km/h and at 5% slope as previously described [32].

Before imaging, the hair was shaved from the 3rd to the 5th right intercostal space just caudal to the right triceps muscle mass, from 3 to 5 cm below the right olecranon to 5 to 10 cm above it. The shaved area was then copiously rinsed with water and acoustic coupling was obtained using ultrasound gel. First repeatability and variability of the technique were tested at rest and the echocardiographic protocol was repeated three times at one-day intervals by the same observer (AAL) on the ten goats. After this study of repeatability, the same echocardiographic protocol was performed on seven of the goats by the same observer (AAL) immediately before and after a standardized exercise on treadmill. The exercise stress echocardiography was performed between 1 week and 4 weeks after the repeatability study. Then, MI was induced in five of the goats. These goats were anaesthetised with 0.3 mg/kg BW of midazolam 5 mg/3 ml (Dormicum, Roche, Bruxelles, Belgium) and 10 mg/kg BW of ketamine 50 mg/ml (Ketalar, Pfizer, Bruxelles, Belgium) using a catheter in the right jugular vein. Tracheal intubation was performed using a 9.0 mm or 10.0 mm ID endotracheal tube and the goats were ventilated with a tidal volume of 15 ml/kg BW with a respirator using a mixture of 2% isoflurane in oxygen. The left common carotid artery (*arteria carotis communis*) was punctured and canulated with a sterile 6 French introducer sheath (Cook Medical, Bloomington, IN, USA) to allow introduction of a Judkins left 6 French catheter (Medtronic, Heerlen, Pays-Bas) under cine-fluoroscopic guidance (Pulsera, Philips, Eindhoven, Pays-Bas) into the right and left coronary trunks. As soon as the coronary branches from the left coronary artery were identified, infarction was performed using a coil embolization. A microguide (Cook Medical, Bloomington, IN, USA) was advanced in the left circumflex coronary artery (*ramus circumflexus sinister*) and placed into the left marginal branch of the left circumflex artery (*ramus marginis ventricularis sinister*), into the posterior descending branch of the left circumflex artery (*ramus interventricularis subsinuosus*), or just upstream from these two vessels. A coil delivering microcatheter (Cook Medical, Bloomington, IN, USA) was placed into the selected location and a platinum Nester embolization microcoil 30x2mm or 30x3mm (Cook Medical, Bloomington, IN, USA) was pushed into the vessel using a wire coil pusher (Cook Medical, Bloomington, IN, USA). Ten minutes after coil embolization, total coronary occlusion of the left marginal branch (in 1 goat) or of the posterior descending branch (in 2 goats) or of the left circumflex artery just upstream from these two vessels (in 2 goats) was confirmed by angiography. When cardiopulmonary status of the goat was stable, the left common carotid artery, the subcutaneous tissue and the skin were sutured. Then the goat was awakened in sternal position and endotracheal tube was removed as soon as a gag reflex was present. After a 24 h post-operative intensive care follow-up including continuous ECG and pain management with intravenous administration of 1.1 mg/kg BW of flunixin (Finadyne, Intervet, Bruxelles, Belgium), 2DST echocardiography was performed on unanaesthetised standing goats to assess average and regional myocardial function. At the end of the experiments, all goats were deeply sedated with intravenous administration of xylazine 0.2 mg/kg BW (Proxylaz, Prodivet, Eynatten, Belgium) then euthanized with intravenous administration of 10 ml of an association of embutramide 200 mg/ml, mebezonium iodide 50 mg/ml and tetracaine hydrochloride 5 mg/ml (T 61, Intervet, Bruxelles, Belgium).

### Echocardiographic examination

An ultrasound system (Vivid i, Software version 9.1.0, General Electric Healthcare Europe GmbH, Diegem, Belgium) equipped with a 1.5–3.6 MHz phased array transducer (GE 3S-RS probe, General Electric Healthcare Europe GmbH, Diegem, Belgium) was used to perform the echocardiography. All examinations were performed by the same observer (AAL) on standing animals with the right forelimb extended by an assistant as far forward as tolerated by the goat. Echocardiographic images were recorded digitally as cine-loops. An imaging depth between 11 and 15 cm, and a frame rate between 54 and 115 frames/second in 2D-mode, was used for all examinations. 2D image terminology and orientation recommended by the Echocardiography Committee of The Specialty of Cardiology, American College of Veterinary Internal Medicine, were used [38]. At each examination, a right parasternal long axis four chambers view with chordae tendinae and mitral valve clearly visible was obtained in 2D-mode. From this view, the transducer was turned clockwise until obtaining a 2D-mode right parasternal short-axis view of the left ventricle at the level of the papillary muscles and several cine-loops of this view were recorded. Care was taken to avoid oblique views and to allow good endocardial delineation.

### Offline 2DST analysis

All 2DST analyses were performed blindly, in a random order, by the same observer (AAL) using the 2D strain application of a specific software (Echo Pac System for Vivid i, Software version 108.1.5, General Electric Healthcare Europe GmbH, Diegem, Belgium) as previously described by several authors [14–16, 19, 20]. All variables were measured five times on five different non consecutive cycles. For each measurement, a 2D-mode right parasternal short-axis cine-loop during one cardiac cycle and with an optimal image quality was selected and the semi-automated 2D strain application was started using the SAX-PM option. The ROI were manually traced starting at the anterior interventricular septum and following the endocardial border in a clockwise manner. After careful endocardial border definition, 2D-strain application was launched and enabled an automated radial and circumferential LV strain analysis. The software divided the myocardium into six segments according to the human guidelines (Ant: anterior; Antsept: anteroseptal; Inf: inferior; Lat: lateral; Post: posterior; Sept: septal), and allowed assessment of the tracking quality (Fig. 1). After the operator has visually verified the tracking, the analysis was approved and six curves, each corresponding to each ROI segment, were analyzed. Peak strain and strain rate measurements were performed automatically by the software and provided in a table. Circumferential strain (Sc), radial strain (Sr), circumferential strain rate (SRc), radial strain rate (SRr), radial displacement (Dr), rotation (Rot) and rotation rate (RotR) were measured. For each variable one or several peaks were measured for each ROI segment. Each peak value was automatically calculated by the program then manually adapted when needed. Measurements for each ROI segment included: one systolic peak for Sc and Sr (Sc-S and Sr-S); 3 peaks for SRc, SRr and RotR, comprising one systolic peak (SRc-S, SRr-S and RotR-S), one early diastolic peak (SRc-E, SRr-E and RotR-E) and one late diastolic peak (SRc-A, SRr-A and RotR-A); and one single systolic peak for DR and Rot (DR-S and Rot-S) (Fig. 2). The average peak values of each 2DST variables corresponding to the mean of the 6 ROI segments measurements was calculated manually. The time of the aortic valve closure was automatically calculated by the software since this calculation was demonstrated to be reliable in goats for 2DST short axis analyses at the papillary muscle level [20].

Heart rate (HR) was calculated from the electrocardiograms from five cineloops containing five successive cardiac cycles and including the ones used for the offline measurements. Echocardiographic recordings at rest were only measured when HR was below 110 bpm. Exercise stress echocardiography was performed immediately after the standardized treadmill exercise test, images were recorded within 2 min after exercise cessation and measurements after exercise were performed only when HR was between 130 and 140 bpm.

### Statistical analysis

Statistical analyses were performed using a computer statistical software (Statistical Analysis System, version 9.1, SAS Institute Inc., Cary, NC, USA) and a standard computer software (Microsoft Office Excel 2003, Microsoft corp, Redmond, WA, USA). The repeatability of the measurements was established by analysis of variance with respect to goat and time factors (two-way ANOVA) and by calculating coefficients of variation both between cycles and between days for each parameter. A two-way ANOVA considering goats, days and interaction between goats and days as factors, allowed the determination of between-day differences of the measurements. Moreover observed means, standard deviations (SD), least square means, and standard errors (SE), were automatically calculated for each variable and each day. The within-goat within-day between-cycle variability was evaluated using the coefficient of variation (Between-cycle CV) measured from SD and observed means obtained from the two-way ANOVA for each variable. The within-goat between-day variability (Between-day CV), representing the variability of the same repeated measurements on the same goat, without taking into account the day of examination (i.e. the measurements of each parameters for each goat were pooled without considering the day of the measurements), was measured from SD and observed means obtained in a one-way ANOVA considering only goats as factor. Degree of variability of each measurement was defined as applied in previous studies [16, 19]: variables with a CV inferior to 15% were considered to have low variability, those with a CV between 15 and 25% were considered to have moderate variability, and those with a CV superior to 25% were considered to have high variability. In addition to the CV, absolute variability was obtained by calculating the interval of confidence of the mean for each variable. This interval of confidence was defined as the interval within the absolute value of the mean of the studied population had 95% of probability to be included. Superior and inferior limits of this interval were calculated as follows: observed mean + 2.262 x SE and observed mean – 2.262 x SE. Measurements were considered repeatable if both a non-significant result of the two-way ANOVA and a low or moderate variability were observed.

Concerning stress echocardiography analysis, observed means, SD, least square means, and SE were calculated for each variable before and after exercise. A two-way ANOVA considering goats, exercise, and interaction between goats and exercise as factors, allowed the comparison between measurements obtained at rest and immediately after exercise.

One to 3 days after stress-echocardiography, five of the studied goats underwent the experimental model of ischemic cardiomyopathy procedure. Echocardiography performed at rest before exercise test was considered as baseline. Then the same echocardiographic protocol was performed on the five goats by the same observer at 24 h after MI induction and each variable was measured five times on five different non consecutive cardiac cycles. Observed means, SD, least square means, and SE were calculated for each variable. A two-way ANOVA considering goats, MI, and interaction between goats and MI as factors, allowed the comparison between variable obtained during baseline and after MI creation.

For all statistical analyses, a *P*-value inferior to 0.05 was considered significant.

## Data Availability

The datasets used and/or analysed during the current study are available from the corresponding author on reasonable request.

## Abbreviations

2DST: Two-dimensional speckle tracking
-A: Measurement during the atrial contraction in diastole
Ant: Anterior
Antsept: Anteroseptal
CI: Confidence intervals
CV: Coefficient of variation
Dr: Radial displacement
-E: Measurement during the early ventricular filling in diastole
Inf: Inferior
Lat: Lateral
LV: Left ventricular
MI: Myocardial infarction
Post: Posterior
ROI: Region of interest
Rot: Rotation
RotR: Rotation rate
-S: Measurement in systole
Sc: Circumferential strain
SD: Standard deviation
SE: Standard error
Sept: Septal
Sr: Radial strain
SRc: Circumferential strain rate
SRr: Radial strain rate
